# Interdisciplinary decision making in prostate cancer therapy – 5-years’ time trends at the Interdisciplinary Prostate Cancer Center (IPC) of the Charité Berlin

**DOI:** 10.1186/1472-6947-13-83

**Published:** 2013-08-05

**Authors:** Daniel Baumunk, Roman Reunkoff, Julien Kushner, Alexandra Baumunk, Carsten Kempkensteffen, Ursula Steiner, Steffen Weikert, Lutz Moser, Mark Schrader, Stefan Höcht, Thomas Wiegel, Kurt Miller, Martin Schostak

**Affiliations:** 1 Department of Urology and Pediatric Urology, Magdeburg University Medical Center, Leipziger Strasse 44, Magdeburg, D-39120, Germany; 2 Department of Urology, Bethanien Medical Center Chemnitz, Zeisigwaldstr. 101, Chemnitz D-09130, Germany; 3 Department of Urology, Charité-Berlin University Medical Center, Charitéplatz 1, Berlin D-10117, Germany; 4 Department of Radiation Oncology, Charité-Berlin University Medical Center, Hindenburgdamm 30, Berlin D-12200, Germany; 5 Department of Urology, Ulm University Medical Center, Prittwitzstrasse 43, Ulm D-89075, Germany; 6 XCare-Radiation Oncology at Marienkrankenhaus Medical Center, Kapuziner Straße 4, Saarlouis 66740, Germany; 7 Department of Radiation Oncology, Ulm University Medical Center, Prittwitzstrasse 43, Ulm D-89075, Germany

**Keywords:** Prostate cancer, Interdisciplinary consultation, Medical decision making, Time trends

## Abstract

**Background:**

Patients with prostate cancer face the difficult decision between a wide range of therapeutic options. These men require elaborate information about their individual risk profile and the therapeutic strategies´ risks and benefits to choose the best possible option. In order to detect time trends and quality improvements between an early patient population (2003/2004) and a later reference group (2007/2008) data was analysed with regards to epidemiologic parameters, differences in diagnostics and the type and ranking of the recommended therapies taking into account changes to Gleason Grading System and implementation of new therapeutic strategies, particularly Active surveillance, in 2005.

**Methods:**

Data from all 496 consecutive patients who received consultation in 2003/2004 (n = 280) and 2007/2008 (n = 216) was retrospectively evaluated. Categorical variables were compared using the Chi-square test. Dependent variables were analysed using the unpaired Students´ t-test and the Mann–Whitney U-test.

**Results:**

The cohorts were comparable concerning clinical stage, initial PSA, prostate volume, comorbidities and organ confined disease. Patients in Cohort I were younger (66.44 vs. 69.31y; p < .001) and had a longer life expectancy (17.22 vs. 14.75y; p < .001). 50.9%, 28.2% and 20.9% in Cohort I and 37.2%, 39.6% and 23.2% in Cohort II showed low-, intermediate- and high-risk disease (D´Amico) with a trend towards an increased risk profile in Cohort II (p = .066). The risk-adapted therapy recommended as first option was radical prostatectomy for 91.5% in Cohort I and 69.7% in Cohort II, radiation therapy for 83.7% in Cohort I and 50.7% in Cohort II, and other therapies (brachytherapy, Active surveillance, Watchful waiting, high-intensity focused ultrasound) for 6.5% in Cohort I and 6.9% in Cohort II (p < .001). Radiation therapy was predominant in both cohorts as second treatment option (p < .001). Time trends showing quality improvement involved an increase in biopsy cores (9.95 ± 2.38 vs. 8.43 ± 2.29; p < .001) and an increased recommendation for bilateral nerve sparing (p < .001).

**Conclusion:**

In the earlier years, younger patients with a more favourable risk profile presented for interdisciplinary consultation. A unilateral recommendation for radical prostatectomy and radiation therapy was predominant. In the later years, the patient population was considerably older. However, this group may have benefitted from optimised diagnostic possibilities and a wider range of treatment options.

## Background

At present, about 63.000 new cases of prostate cancer (PCa) are diagnosed every year in Germany [1]. Increasing knowledge regarding the heterogeneity of PCa [2,3] and its variable clinical course has sparked controversy over the best treatment approach [4-6]. Between diagnostic and therapeutic uncertainty on the one hand, and overtreatment on the other hand, physicians have an important obligation to provide patients with complete information on treatment options and their side effects [7]. The patient finds himself confronted with various treatment options [8-11], and his choice of treatment may also be substantially influenced by other factors such as family considerations, social environment, social status, and the patient-consultant relationship [12]. Many patients favour shared medical decision making [13]. The usually long clinical course of PCa complicates the choice of treatment. Comorbidity evaluation also plays an important role in this context [14].

Since 2001, interdisciplinary consultation then supported by the German Cancer Aid (until 2006; project number 70–2945) has been provided to approximately 2500 PCa patients by experienced urologists and radiation therapists at the IPC [7]. The patients (and family members) are informed face to face by the urologist and radiation oncologist. Consultation contains a full overview of the possible therapeutic options with regard to the information about the individual risk profile: patient’s age and comorbidities, medication, palpation findings, PSA level, Gleason Score of biopsy, number of positive biopsy cores, CT- or MRI scan, as far as known at the time of consultation. The duration of the consultation is variable and depends on the individual demands of the patient or family members and varies averagely between 15 to 60 minutes.

Important changes and new complements in prostate cancer therapy around the year 2005 like the revision of the Gleason Grading System [15], the implementation of Active surveillance (AS) as a treatment option for low-risk cancers and non-standardised therapeutic alternatives like high-intensity focussed ultrasound (HIFU), cryotherapy or laser-based ablation techniques, have led to a greater variety of treatment options. In order to prove an impact of these changes and complementary therapeutic options as well as to evaluate the quality of our interdisciplinary consultation, time trends between an early patient population (2003/2004) and a later reference group (2007/2008) were analysed with regard to epidemiologic factors and pre-clinical diagnostics as well as changes in the type and ranking of treatment recommendations.

## Methods

Data from all 496 consecutive patients who received interdisciplinary consultation in 2003/2004 (Cohort I; n = 280) and 2007/2008 (Cohort II; n = 216) could be analysed. The patients presenting for consultation had to a great extent newly diagnosed, localised prostate cancer. The majority of patients was diagnosed pre-clinically by registered urologists and was then presented to the IPC for interdisciplinary consultation. Also patients with advanced prostate cancers presented for consultation in terms of a second opinion. As the patients presented for a medical consultation with no additional invasive medical procedures (e. g. additional blood samples etc.) and not within the context of a clinical or experimental study, no special ethical approvement was required (Ethics Review Committee of the Charité-Berlin University Medical Center; EA4/082/13). All patients gave their written informed consent for the evaluation and publication of the anonymised data generated within the context of the consultation. Analysis of the data encompassed important epidemiological parameters as far as known at the time of consultation (clinical stage, PSA at the time of diagnosis, biopsy Gleason score, D’Amico risk group stratification, prediction of prognosis according to Partin and Kattan, and Charlson comorbidity score) as well as the type and ranking of the recommended therapies. The recommended therapies were: radical prostatectomy (RP; open surgery, conventional laparoscopic surgery, and robot-assisted laparoscopic surgery), external 3D-CT-planned external beam radiation therapy (EBRT) with at least 73.8 Gy or IMRT, brachytherapy (BT; Seeds or HDR brachytherapy), Active surveillance (AS; since 2005), Watchful waiting (WW), high-intensity focused ultrasound (HIFU; since 2005), and androgen deprivation (AD). For better presentation and greater clarity, the focus lies on the first two ranks of recommendation. Equally valid recommendations were given the same ranking.

Over the years, patients received consultation from a total of 17 urology specialists and 10 radiation oncology specialists. Data was assessed on the basis of medical records and by questionnaires when necessary. Normal distribution was verified by the Kolmogorov-Smirnov test. Data analysis was done using SPSS software version 19.0 (SPSS Inc., Chicago Il, USA). Categorical variables were compared using the Chi-square test. The unpaired Students´ t-test and the Mann–Whitney U-test were used for dependent variables as appropriate. The significance level was set at .05.

## Results

### Epidemiology

PCa diagnosis was based on an elevated serum PSA level for 86.8% of the patients in Cohort I and 88.9% of the patients in Cohort II, suspicious palpation findings for 25% of the patients in Cohort I and 13.4% of the patients in Cohort II, histology after transurethral resection of the prostate (TURP) for 2.5% in Cohort I and 4.6% in Cohort II, and PCA-associated symptoms for 7.1% in Cohort I and 10.6% in Cohort II with no measurable group differences (p > .05). Table 1 summarises the symptoms or diagnostic findings which led to PCa diagnosis.

**Table 1:** Symptoms or diagnostic findings leading to prostate cancer diagnosis

	**Cohort I**	**Cohort II**	p-value*
	***(%)***	***(%)***	
**Elevated PSA levels**	*86.8*	*88.9*	*>.05*
**Suspicious palpation**	*25*	*13.4*	
**Histology after TURP**	*2.5*	*4.6*	
**PCa associated symptoms**	*7.1*	*10.6*	

*Student´s T-test; Symptoms or diagnostic findings leading to PCa diagnosis: elevated PSA levels, suspicious palpation of the prostate, incidental histology after transurethral resection of the prostate (TURP) and PCa associated symptoms.

At the time of interdisciplinary consultation, analysis of the mean age of patients disclosed marked group differences: 66.44 years in Cohort I vs. 69.31 years in Cohort II (p < .001). The mean life expectancy was 17.22 years in Cohort I and 14.75 years in Cohort II (p < .001). 70.9% of the patients in Cohort I and only 40.8% of the patients in Cohort II had a life expectancy of more than 15 years (p < .001).

The median PSA value at the time of diagnosis was 7.98 ± 22.59 μg/l (0.4-311) in Cohort I and 7.6 ± 15.66 μg/l (0.88-120) in Cohort II with no group differences (p = .392).

Analysis of the number of cores in the diagnostic prostate biopsy showed a significant group difference in favour of Cohort II: 9.95 ± 2.38 (2–20) vs. 8.43 ± 2.29 (2–14) in Cohort I; p < .001. The two cohorts did not differ with regard to the prostate biopsy sites or the mean prostate volume (40.16 ml ± 15.64 in Cohort I and 41.75 ml ± 19.56 (n = 66) in Cohort II; p = .689). The biopsy Gleason score was 6 ± 1.43 in Cohort I and 6.59 ± 0.99 (p < .001) in Cohort II.

The clinical stages were distributed as follows: T1a-c 66.1% in Cohort I and 74.4% in Cohort II, T2a-c 28.8% in Cohort I and 22.3% in Cohort II, T3 1.1% in Cohort I 0.5% in Cohort II and T4 0.7% in Cohort I and 0.5% in Cohort II (missing: 3.3% in Cohort I and 2.3% in Cohort II; p = .602).

There were no group differences concerning accompanying diseases measured by the Charlson comorbidity score (CCS): 0.41 (0–6) in Cohort I and 0.39 (0–6) in Cohort II (p = .787).

The groups were also comparable in terms of organ-confined disease (T2) according to Partin Tables (2007) (Cohort I: 61.12%; Cohort II: 58.98%; p = .324). The Kattan nomogram predicted a 5-year biochemical failure free survival of 79.79% in Cohort I and 79.16% in Cohort II (p = .735) after RP and a rate of 79.27% (Cohort I) and 79.77% (Cohort II; p = .657) after EBRT with no group differences.

Patients were stratified by D’Amico classification into low-, intermediate- and high-risk groups as follows: 50.9%, 28.2% and 20.9% in Cohort I; 37.2%, 39.6% and 23.2% in Cohort II, showing a trend towards an increased risk profile in Cohort II (p = .066).

In Cohort I, 129/280 patients (46%) underwent preoperative bone scan, which was positive in 17 cases (13.2%). If the current national or international guidelines [8,10,11] had been applied at that time, 120 of the 129 (93%) bone scans would have been indicated. In Cohort II, bone scan was performed in 75/216 patients (34.7%) and was positive in 3 cases (4%). Based on the current national- and international guidelines [8,10,11], bone scan would have been indicated for 91 patients in Cohort II.

At the time of consultation, 143/261 (54.7%) patients in Cohort I and 86/191 (45%) in Cohort II were potent in terms of erections adequate for sexual intercourse (IIEF-5-Score ≥20). In this group of potent patients, the preservation of potency was important to 85.3% in Cohort I and to 87.2% in Cohort II.

Table 2 summarises the epidemiological and clinical parameters.

**Table 2:** Epidemiologic parameters at the time of consultation

	**Cohort I**	**Cohort II**	p-value*
	***(SD and/or range)***	***(SD and/or range)***	
**Age***(mean)*	*66.44*	*69.31*	*.001*
**Life expectancy***(mean)*	*17.22*	*14.75*	*.001*
>15 y *(%)*	*70.9*	*40.8*	
**Clinical stage***(%)*			
T1a-c	*66.1*	*74.4*	*.602*
T2a-c	*28.8*	*22.3*	
T3	*1.1*	*0.5*	
T4	*0.7*	*0.5*	
**PSA value** *(*μg/l*)*	*7.98 ± 22.59 (0.4-311)*	*7.6 ± 15.66 (0.88-120)*	*.392*
**Organ confined** (Partin; *mean; %*)	*61.1*	*58.9*	*.324*
**Risk groups** (D´Amico; *%)*			
low-risk	*50.9*	*37.2*	*.066*
intermediate risk	*28.2*	*39.6*	
high risk	*20.9*	*23.2*	
**Charlson comorbidity score**	*0.41 (0–6)*	*0.39 (0–6)*	*.787*
**Biochemical failure free survival** (Kattan; *%*)			
RP	*79.8*	*79.2*	*.735*
EBRT	*79.3*	*79.8*	*.657*
**Gleason score of biopsy***(median)*	*6 ± 1.43*	*6.59 ± 0.99*	*<.001*
**Number of biopsy cores**	*8.43 ± 2.29 (2–14)*	*9.95 ± 2.38 (2–20)*	*<.001*
**Prostate volume***(ml)*	*40.16 ± 15.64*	*41.75 ml ± 19.56*	.*689*

*Student´s T-test and Chi-Square-test as appropriate; Epidemiologic parameters at the time of consultation; mean age in years; mean life expectancy and percentage of patients with life expectancy >15 years; clinical stage (in %; missing clinical stage: 3.3% in Cohort I and 2.3% in Cohort II); PSA value in μg/l; percentage of organ confined disease according to Partin 2007; risk group stratification according to D´Amico (in %); Charlson comorbidity score; 5-years biochemical failure free survival according to Kattan nomogram (in %); median Gleason score of biopsy; number of biopsy cores; prostate volume in ml.

### Treatment recommendations and ranking

In Cohort I, the D’Amico risk-adapted therapy recommended as the first treatment option was RP for 91.5%, EBRT for 83.7%, BT for 2.4%, and AD for 3.3% (p < .001). The overlap in patients who received the equally valid recommendation for RP and EBRT was 33.9%. Predominant in the second rating of recommendations in Cohort I was EBRT (16%), followed by RP (8.5%; p < .001).

**Figure 1:**
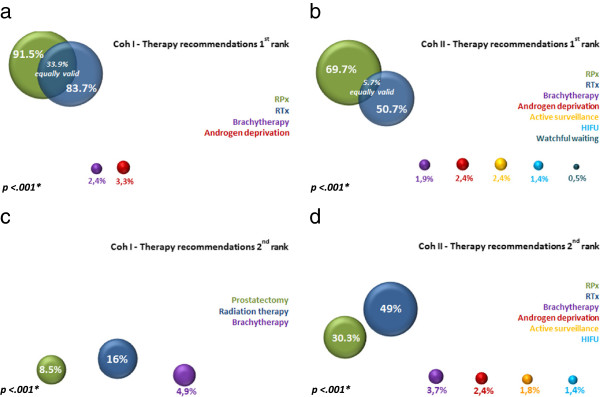
**Therapy recommendations in the first and second rank** green = Prostatectomy/ RPx; blue = EBRT/ RTx/Radiation therapy; violet = Brachytherapy; red = Androgen deprivation; light orange = Active surveillance; light blue = HIFU, petrol blue = Watchful waiting. **a)** Therapy recommendations in Cohort I in the first rank (in percent (%)); *p-value calculated as Chi-Square-test; Intersections of equally valid recommendations for Prostatectomy and EBRT are specified within the figure. **b)** Therapy recommendations in Cohort II in the first rank (in percent (%)); *p-value calculated as Chi-Square-test; Intersections of equally valid recommendations for Prostatectomy and EBRT are specified within the figure. **c)** Therapy recommendations in Cohort I in the second rank (in percent (%)); *p-value calculated as Chi-Square-test. **d)** Therapy recommendations in Cohort II in the second rank (in percent (%)); *p-value calculated as Chi-Square-test.

In Cohort II, the D’Amico risk-adapted therapy recommended as the first treatment option was RP for 69.7%, EBRT for 50.7%, AD for 2.4%, AS for 2.4%, BT for 1.9%, HIFU for 1.4%, and WW for 0.5% (p < .001). The overlap in patients who received the equally valid recommendation for RP and EBRT was 5.7%. Predominant in the second rating of recommendations in Cohort II was again EBRT (49%), followed by RP (30.3%), BT (3.7%), AD (2.4%), AS (1.8%), and HIFU (1.4%; p < .001). The rankings of recommendations are shown in Figure 1.

**Figure 2:**
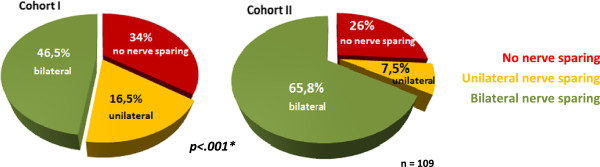
**Recommendation for nerve sparing in patients with ´radical prostatectomy´ as first rank therapy recommendation.** *Chi-Square-test; Nerve sparing recommendation in patients with ´radical prostatectomy´ as first rank therapy recommendation: red = ´no nerve sparing´; yellow = ´unilateral nerve sparing´; green = ´bilateral nerve sparing´.

Cohort I patients with a recommendation for RP had a mean age of 65.8 (50–78) years, Cohort II patients of 67.46 (48–78) years. No nerve sparing was recommended in 34%, unilateral nerve sparing in 16.5% and bilateral nerve sparing in 46.5%. In this context, there was a group difference in favour of Cohort II patients where no nerve sparing was recommended in 26%, unilateral nerve sparing in 7.4% and bilateral nerve sparing in 65.8% (p < .001; Figure 2).

Of the patients with a recommendation for RP, 33 in Cohort I (18%) and 23 in Cohort II (21.7%) were receiving long-term anticoagulant therapy with acetylsalicylic acid, while 4 in Cohort I (10.4%) and 11 in Cohort II (2.1%) were on long-term oral anticoagulation therapy (e. g. phenprocoumon) with a slight trend towards an increased number of recommendations for surgery in patients with anticoagulation therapy in favour of patients in Cohort II (p = .083; not shown).

Patients with a recommendation for EBRT had a mean age of 66.43 (50–81) years in Cohort I and 69.43 years (48–85 years) in Cohort II. Of the patients with a recommendation for EBRT, 44 (21%) in Cohort I and 56 (27.8%) in Cohort II were advised to undergo pelvic lymphadenectomy previous to radiation. Neoadjuvant and/or adjuvant AD was recommended for 71 patients (33.9%) in Cohort I and 66 (32.8%) in Cohort II. BT was recommended for 34 (12.1%) patients in Cohort I and 30 (13.9%) in Cohort II without group differences. There was a slight trend towards favouring EBRT for patients over 70 years of age and RP for patients under the age of 70 (p = .069; not shown).

AS was not considered an option until 2005 and was therefore only recommended for Cohort II patients. A total of 45 patients (20.8%) received the recommendation for AS as a treatment option in the first and second rank of recommendations. Based on the definition of patients eligible for AS given by Klotz et al. [6], AS could have been offered to 55 patients in Cohort II. Given the risk profile of Cohort I, AS could have been recommended for 78 patients (27.8%) in accordance with the national and international guidelines [8,10,11].

AD alone was recommended for 14 patients (5%) in Cohort I and 13 (6%) in Cohort II. WW was recommended as a treatment option for 2 patients in Cohort I (0.7%) and for 3 patients in Cohort II (1.3%). HIFU was recommended for 27 patients (12.5%) since 2005. A recommendation of combined HIFU with pre-HIFU TURP was given to 11 of these 27 patients (40.7%).

## Discussion

This analysis disclosed differences in the age structure of the two cohorts with a corresponding impact on the recommended treatment options. It has confirmed an age shift towards older patients with more widely varying forms of the disease. No significant group differences were found with regard to other epidemiological parameters or comorbidities.

Tumor-specific differences between the groups were particularly difficult to analyse, since the revision of the Gleason grading system in 2005 [15] caused a shift in the risk profile from the majority of low-risk cases in Cohort I to a majority of intermediate-risk cases in Cohort II. This shift was detectable across all risk groups and was mainly due to the upgrading of Gleason score 6 to Gleason score 7a in up to 30%.

### Time trends in the present patient population

Concerning prostate biopsy, there was a difference in the number of biopsy cores taken in favour of the patients in Cohort II. In this context, the increased number of biopsy cores in the later cohort may be associated with the release of recommendations given by the national ´S3-guidelines on PCa´- working group in 2007 [8] in accordance with international guidelines [10] recommending 10 or more biopsy cores. The patients in Cohort II seemed to have received the recommended number of cores which may be interpreted as a sign of quality improvement. On the other hand, taking more cores does not necessarily mean an improvement of quality taking into account, that the patients in Cohort II were considerably older.

Another sign of quality improvement in terms of guideline recommendations was the fact, that bilateral nerve sparing was more frequently recommended to patients in Cohort II.

Our patient population also showed a distinct trend with regard to the increased recommendation for surgery in patients under anticoagulant therapy in favour of Cohort II patients. Treatment with platelet aggregation inhibitors is particularly common in the age group of patients with PCa. Especially in patients with newly-implanted coronary stents, there seems to be consensus that platelet aggregation inhibition should be continued during RP.

As aforementioned there was a measureable difference in the age structure and life expectancy between the two cohorts. Obviously the increased therapeutic options after 2005 may have led to a higher number of patients presenting with more varying forms of the disease or at later stages of the disease. Possibly these patients might have been older than those newly diagnosed with localised stages of the disease. On the other hand, since guidelines have made it easier for registered urologists to advise patients, those patients with clear preferences for one of the treatment options were simply not presented any more for consultation at the IPC. However, as these explanations are speculative, we do not have a sufficient explanation to our observation.

In terms of age and therapy recommendations, we found a trend towards favouring RP for patients under 70 years and EBRT for those over 70 years. This trend could have been a result of the hypothesis at that time, that older patients may have a poorer recovery of continence after surgery. However, in their recently published analysis of 1636 patients, Kunz et al. showed that patients over the age of 70 do not face higher surgical risks and that they are not even at higher risk for postoperative incontinence. Instead, surgery-associated morbidity was mainly dependent on the patients’ preexisting comorbidities [16].

Marked group differences were found especially in the first rank of therapy recommendations. In the earlier Cohort I, the standard therapies were predominant. Cohort II showed a marked percentage decrease in recommendations for RP and EBRT as first treatment option. One simple explanation of this observation might be again that fewer patients with a low-risk profile and an obvious preferation for one of the standard therapies presented for consultation. Presumably, the decrease in recommendation for RP or EBRT in the later cohort was due to the above-mentioned shifts in the patients’ age structure and risk profile and because of the introduction of AS and HIFU as new treatment options in 2005. Therefore, the patients in the later cohort II may have benefitted from the greater variety of treatment options.

In both cohorts, EBRT was recommended most often in the second rank of recommendations.

AS according to the commonly applied specifications of Klotz et al. [6] was recommended for 20.8% of the patients in the later Cohort II in any rank. An even higher proportion of the Cohort I patients (27.8%) would have qualified for AS, if it had been available as a treatment option at that time. It is interesting to note that AS as a ´no treatment´- or probably ´delayed treatment´ strategy was the recommendation for as many as one-fifth of patients with PCa. In this respect, PCa probably has a top position among tumor entities, but still there are diagnostic difficulties and uncertainties in the risk stratification of these patients [17].

AD and WW were a first-rating recommendation in basically only a few cases.

An interdisciplinary shared decision making-process as presented here is time-consuming. However, providing a detailed description of all treatment options leads to high levels of patient satisfaction [7,18]. Apart from that, comprehensive specialist consultation is of great importance against the background of the current discussions regarding overtreatment-induced side effects and high costs on the one hand and the potential consequences and uncertainty as well as additional expenses of delayed treatment on the other hand.

A face to face setting with the patient is ideal to work out the patient´s preferences. Meghani et al. recently showed that men with prostate cancer may be divided into subgroups with some men being likely to trade survival time to prevent potential treatment risks while others may accept treatment risks and are unlikely to trade survival time [19]. The vast majority of cancer patients of either gender desire maximal information. It was demonstrated, however, that a significantly higher proportion of those who did not want maximal information were over the age of 70 [20]. There is considerable individual variation in the amount of information required during consultation. However, patients attach importance to receiving individual consultation and sharing treatment decisions [21,22]. Men with PCa expect the physician to explain the diagnosis and describe the treatment options. Discussing these expectations at the beginning of a consultation session can lead to a satisfactory consensus decision [23]. Decision making on the basis of the best possible understanding enables patients to realistically appraise their personal risk profile and the potential side effects of therapy [24]. In an analysis of 3056 PCa patients, Resnick et al. showed that the satisfaction of patients with their PCa treatment was not negatively influenced to any great extent by the side effects of the therapy recommended [25].

A communication mistake can entail overloading patients with too much information [26]. Providing too little information is a far more common error, however. This is the main cause of a decisional conflict in those affected [27]. In men under AS, Goh et al. found treatment decision satisfaction particularly among patients who perceived themselves as being well-informed and therefore felt they had control over their disease and symptoms [28]. The most important factor affecting the treatment decision of PCa patients is the physician [29-31]. Apart from family considerations, negative experiences of other cancer patients also exert an important influence on the treatment decision [30]. Denberg et al. showed that the treatment decision of many patients was determined by misconceptions and bad experiences of other patients. Most patients did not change their beliefs and judged the success of therapy in terms of the same misconceptions [32]. In this context patients´ anxiety about the disease is widely underestimated. Efforts are made to measure the influence of patients´ anxiety on decision making in prostate cancer [33].

Despite the high number of cases and data density, there is a lack of relevant information at the time of consultation, particularly from the earlier years (Cohort I). Missing information includes standardised quality-of-life assessment (e. g. EORTC QLQ-C30, QLQ-PR25) and evaluation of micturition (IPSS), erectile function (IIEF) and continence (ICIQ-SF), which are considered standard in the pre- and post-therapeutic situation [34,35]. These data have been regularly collected since 2005. However, no time trends can be investigated in this analysis due to the inadequate data collection in Cohort I. Different limitations to the data provided lie in the unicenter data collection and the lack of long-term clinical courses. Since consultation was basically offered to all patients, data on the treatment actually implemented are not available in a relevant proportion of those who presented for consultation but were not treated at the IPC. Thus no statement can be made at present regarding congruence between the recommended and the implemented treatment. Strong efforts are currently being directed towards generating these data. Another factor limiting the quality of consultation is the large number of physicians involved over the years. Even though guideline specifications were continuously integrated into the working procedures in regular quality circles of the IPC, consultation quality still remains dependent on the knowledge and conversation techniques of the consultant.

Individualisation of PCa treatment is a reality. Nevertheless, the value of the various treatment options has not yet been prospectively assessed in a multicenter study. A study aimed at clarifying this question for patients in a low-risk situation of the disease is the oncoming ´PREFERE´ trial [36]. Prospective comparative evaluation of the effectiveness of the various treatment options is necessary in order to prevent harm being done to patients. Apart from enhanced transmission of information, improvements in imaging [37] and advanced diagnostic procedures like the analysis of circulating tumor cells [38] will lead to further individualisation of the treatment options. Learning programs like PIES (Prostate Interactive Educational System) and other similar decision aids may provide standardised information and objective decision support for patients, family members and physicians [39,40]. However these print- or web-based decision tools may not be useful for every patient and need further improvements [41]. Decision aids may increase patients´ perception of the desease but it is not guaranteed that they lead to an increase of shared decision making [42]. Last but not least, the possibility of free online access to information about studies such as the ´Australian Cancer Trials´ [43] helps to optimise the information basis for patients, family members and physicians.

## Conclusion

Our analysis disclosed time trends between the two cohorts. In the early years of interdisciplinary prostate cancer consultation, younger patients with a considerably longer life expectancy presented for consultation and a unilateral recommendation for the standard therapies was therefore predominant. In the later years, there was a greater variety of treatment recommendations due to new therapeutic options like AS and HIFU. Patients in the later cohort may have benefitted from these additional treatment options, which, among other influences, may have reduced the number of recommendations for the standard therapies. Quality improvements over the years were seen in the greater number of biopsy cores in accordance with the recommendations of the national and international guidelines and the increased number of recommendations for bilateral nerve sparing prostatectomy in Cohort II. An interdisciplinary consultation for patients with newly diagnosed PCa is the gold standard with regard to comprehensive patient information.

## Abbreviations

PCa: Prostate cancer; IPC: Interdisciplinary Prostate Cancer Center of the Charité-Universitätsmedizin Berlin, Germany; RP: Radical prostatectomy; EBRT: External beam radiation therapy; BT: Brachytherapy; AS: Active surveillance; WW: Watchful waiting; HIFU: High-intensity focussed ultrasound; AD: Androgen deprivation; TURP: Transurethral resection of the prostate; CCS: Charlson comorbidity score; IPSS: International Prostate Symptom Score; IIEF-5: International Index of Erectile Function; ICIQ-SF: International Consultation on Incontinence-Short Form; QLQ-C30: EORTC Quality of Life Questionnaire - Core Questionnaire; QLQ-PR25: EORTC Quality of Life Questionnaire - Prostate Module.

## Competing interests

The authors declare that they have no competing interests.

## Authors’ contributions

DB participated in the conception, analysed and interpreted the data, wrote the manuscript and gave final approval to the content. RR and JK acquired the data. AB acquired the data and critically revised the manuscript. CK and US participated in the conception and revised the manuscript. SW analysed and interpreted the data and critically revised the manuscript. LM participated in the conception and acquired the data. MS, SH and TW made substantial contributions to the conception of the consultations´ setting. KM critically revised the manuscript. MSC made substantial contributions to the conception and data analysis and -interpretation and critically revised the manuscript. All authors read and approved the final manuscript.

## Pre-publication history

The pre-publication history for this paper can be accessed here:

http://www.biomedcentral.com/1472-6947/13/83/prepub

## References

[B1] GEKID Cancer in Germany 2007/2008, Version 8 2012 Berlin, Germany: Robert-Koch-Institute and the Association of Population-based Cancer Registries in Germany (GEKID)

[B2] Ruijter ET van de Kaa CA Schalken JA Debruyne FM Ruiter DJ Histological grade heterogeneity in multifocal prostate cancer. Biological and clinical implications J Pathol 1996 180 3 295 299 10.1002/(SICI)1096-9896(199611)180:3<295::AID-PATH663>3.0.CO;2-W 8958808

[B3] Macintosh CA Stower M Reid N Maitland NJ Precise microdissection of human prostate cancers reveals genotypic heterogeneity Cancer Res 1998 58 1 23 28 9426051

[B4] Kollmeier MA Zelefsky MJ How to select the optimal therapy for early-stage prostate cancer Crit Rev Oncol Hematol 2012 83 2 225 234 10.1016/j.critrevonc.2011.11.001 22154187

[B5] Bastian PJ Carter BH Bjartell A Seitz M Stanislaus P Montorsi F Stief CG Schroder F Insignificant prostate cancer and active surveillance: from definition to clinical implications Eur Urol 2009 55 6 1321 1330 10.1016/j.eururo.2009.02.028 19286302

[B6] Klotz L Active surveillance for prostate cancer: for whom? J Clin Oncol 2005 23 32 8165 8169 10.1200/JCO.2005.03.3134 16278468

[B7] Schostak M Wiegel T Muller M Hoecht S Schrader M Straub B Bottke D Hinkelbein W Miller K Shared decision-making–results from an interdisciplinary consulting service for prostate cancer World J Urol 2004 22 6 441 448 10.1007/s00345-004-0447-3 15378335

[B8] DGU Interdisciplinary S3-guidelines on the early detection, diagnostics and therapy of the different stages of prostate cancer; AWMF registration number (034-022OL), Version 2 2011 Düsseldorf, Germany: Deutsche Gesellschaft für Urologie e. V. (German Urological Association)

[B9] Tunn UW Advanced prostate cancer treatment guidelines: European perspective BJU Int 2004 94 Suppl 3 6 15521884 10.1111/j.1464-410X.2004.05136.x

[B10] Heidenreich A Bellmunt J Bolla M Joniau S Mason M Matveev V Mottet N Schmid HP van der Kwast T Wiegel T [EAU guidelines on prostate cancer. Part I: screening, diagnosis, and treatment of clinically localised disease] Actas Urol Esp 2011 35 9 501 514 21757259 10.1016/j.acuro.2011.04.004

[B11] Cookson MS Aus G Burnett AL Canby-Hagino ED D'Amico AV Dmochowski RR Eton DT Forman JD Goldenberg SL Hernandez J Variation in the definition of biochemical recurrence in patients treated for localized prostate cancer: the American Urological Association Prostate Guidelines for Localized Prostate Cancer Update Panel report and recommendations for a standard in the reporting of surgical outcomes J Urol 2007 177 2 540 545 10.1016/j.juro.2006.10.097 17222629

[B12] Lobb EA Gaff CL Meiser B Butow PN Osseiran-Moisson R Hallowell N Attendance of men at the familial cancer clinic: what they value from the consultation Genet Med 2009 11 6 434 440 10.1097/GIM.0b013e3181a18982 19346953

[B13] Gattellari M Butow PN Tattersall MH Sharing decisions in cancer care Soc Sci Med 2001 52 12 1865 1878 10.1016/S0277-9536(00)00303-8 11352412

[B14] Daskivich TJ Chamie K Kwan L Labo J Dash A Greenfield S Litwin MS Improved prediction of long-term, other cause mortality in men with prostate cancer J Urol 2011 186 5 1868 1873 10.1016/j.juro.2011.07.033 21944092

[B15] Epstein JI Allsbrook WC Jr Amin MB Egevad LL The 2005 International Society of Urological Pathology (ISUP) Consensus Conference on Gleason Grading of Prostatic Carcinoma Am J Surg Pathol 2005 29 9 1228 1242 10.1097/01.pas.0000173646.99337.b1 16096414

[B16] Kunz I Musch M Roggenbuck U Klevecka V Kroepfl D Tumour characteristics, oncological and functional outcomes in patients aged ≥ 70 years undergoing radical prostatectomy BJU Int 2013 Mar 111 3 Pt B E24 E29 Epub 2012 Sep 5. PMID: 22947135 10.1111/j.1464-410X.2012.11368.x 22947135

[B17] Thaxton CS Loeb S Roehl KA Kan D Catalona WJ Treatment outcomes of radical prostatectomy in potential candidates for 3 published active surveillance protocols Urol 2010 75 2 414 418 10.1016/j.urology.2009.07.1353 19963249 PMC3072831

[B18] Feldman-Stewart D Brundage MD Van Manen L Svenson O Patient-focussed decision-making in early-stage prostate cancer: insights from a cognitively based decision aid Health Expect 2004 7 2 126 141 10.1111/j.1369-7625.2004.00271.x 15117387 PMC5060228

[B19] Meghani SH Lee CS Hanlon AL Bruner DW Latent class cluster analysis to understand heterogeneity in prostate cancer treatment utilities BMC Med Inform Decis Mak 2009 9 47 10.1186/1472-6947-9-47 19941668 PMC2789058

[B20] Jenkins V Fallowfield L Saul J Information needs of patients with cancer: results from a large study in UK cancer centres Br J Cancer 2001 84 1 48 51 10.1054/bjoc.2000.1573 11139312 PMC2363610

[B21] Feldman-Stewart D Brundage MD Nickel JC MacKillop WJ The information required by patients with early-stage prostate cancer in choosing their treatment BJU Int 2001 87 3 218 223 10.1046/j.1464-410x.2001.02046.x 11167645

[B22] Feldman-Stewart D Brundage MD Tong C Information that affects patients' treatment choices for early stage prostate cancer: a review Can J Urol 2011 18 6 5998 6006 22166326

[B23] Davison BJ Parker PA Goldenberg SL Patients' preferences for communicating a prostate cancer diagnosis and participating in medical decision-making BJU Int 2004 93 1 47 51 10.1111/j.1464-410X.2004.04553.x 14678366

[B24] Baade PD Steginga SK Pinnock CB Aitken JF Communicating prostate cancer risk: what should we be telling our patients? Med J Aust 2005 182 9 472 475 15865593 10.5694/j.1326-5377.2005.tb06790.x

[B25] Resnick MJ Guzzo TJ Cowan JE Knight SJ Carroll PR Penson DF Factors associated with satisfaction with prostate cancer care: results from Cancer of the Prostate Strategic Urologic Research Endeavor (CaPSURE) BJU Int 2013 Feb 111 2 213 220 Epub 2012 Aug 29. PMID: 22928860 10.1111/j.1464-410X.2012.11423.x 22928860 PMC3540193

[B26] Zeliadt SB Ramsey SD Penson DF Hall IJ Ekwueme DU Stroud L Lee JW Why do men choose one treatment over another?: a review of patient decision making for localized prostate cancer Cancer 2006 106 9 1865 1874 10.1002/cncr.21822 16568450

[B27] Snow SL Panton RL Butler LJ Wilke DR Rutledge RD Bell DG Rendon RA Incomplete and inconsistent information provided to men making decisions for treatment of early-stage prostate cancer Urol 2007 69 5 941 945 10.1016/j.urology.2007.01.027 17482939

[B28] Goh AC Kowalkowski MA Bailey DE Jr Kazer MW Knight SJ Latini DM Perception of cancer and inconsistency in medical information are associated with decisional conflict: a pilot study of men with prostate cancer who undergo active surveillance BJU Int 2012 110 2 Pt 2 E50 E56 22145791 10.1111/j.1464-410X.2011.10791.x

[B29] Cohen H Britten N Who decides about prostate cancer treatment? A qualitative study Fam Pract 2003 20 6 724 729 10.1093/fampra/cmg617 14701899

[B30] Steginga SK Occhipinti S Gardiner RA Yaxley J Heathcote P Making decisions about treatment for localized prostate cancer BJU Int 2002 89 3 255 260 10.1046/j.1464-4096.2001.01741.x 11856106

[B31] Sidana A Hernandez DJ Feng Z Partin AW Trock BJ Saha S Epstein JI Treatment decision-making for localized prostate cancer: what younger men choose and why Prostate 2012 72 1 58 64 10.1002/pros.21406 21520163 PMC4612632

[B32] Denberg TD Melhado TV Steiner JF Patient treatment preferences in localized prostate carcinoma: the influence of emotion, misconception, and anecdote Cancer 2006 107 3 620 630 10.1002/cncr.22033 16802287

[B33] Linder SK Swank PR Vernon SW Morgan RO Mullen PD Volk RJ Is a prostate cancer screening anxiety measure invariant across two different samples of age-appropriate men? BMC Med Inform Decis Mak 2012 12 52 10.1186/1472-6947-12-52 22681782 PMC3408324

[B34] Efficace F Bottomley A van Andel G Health related quality of life in prostate carcinoma patients: a systematic review of randomized controlled trials Cancer 2003 97 2 377 388 10.1002/cncr.11065 12518362

[B35] van Andel G Bottomley A Fossa SD Efficace F Coens C Guerif S Kynaston H Gontero P Thalmann G Akdas A An international field study of the EORTC QLQ-PR25: a questionnaire for assessing the health-related quality of life of patients with prostate cancer Eur J Cancer 2008 44 16 2418 2424 10.1016/j.ejca.2008.07.030 18774706

[B36] Stockle M Bussar-Maatz R [Localised prostate cancer: the PREFERE trial] Z Evid Fortbild Qual Gesundhwes 2012 106 5 333 335 discussion 335 10.1016/j.zefq.2012.05.004 22818151

[B37] Hambrock T Hoeks C Hulsbergen-Van De Kaa C Scheenen T Futterer J Bouwense S Van Oort I Schroder F Huisman H Barentsz J Prospective assessment of prostate cancer aggressiveness using 3-T diffusion-weighted magnetic resonance imaging-guided biopsies versus a systematic 10-core transrectal ultrasound prostate biopsy cohort Eur Urol 2012 61 1 177 184 10.1016/j.eururo.2011.08.042 21924545

[B38] Danila DC Fleisher M Scher HI Circulating tumor cells as biomarkers in prostate cancer Clin Cancer Res 2011 17 12 3903 3912 10.1158/1078-0432.CCR-10-2650 21680546 PMC3743247

[B39] Lin HC Wu HC Chang CH Li TC Liang WM Wang JY Development of a real-time clinical decision support system upon the Web MVC-based architecture for prostate cancer treatment BMC Med Inform Decis Mak 2011 11 16 10.1186/1472-6947-11-16 21385459 PMC3068074

[B40] Diefenbach MA Mohamed NE Butz BP Bar-Chama N Stock R Cesaretti J Hassan W Samadi D Hall SJ Acceptability and preliminary feasibility of an internet/CD-ROM-based education and decision program for early-stage prostate cancer patients: randomized pilot study J Med Internet Res 2012 14 1 e6 10.2196/jmir.1891 22246148 PMC3846339

[B41] Dorfman CS Williams RM Kassan EC Red SN Dawson DL Tuong W Parker ER Ohene-Frempong J Davis KM Krist AH The development of a web- and a print-based decision aid for prostate cancer screening BMC Med Inform Decis Mak 2010 10 12 10.1186/1472-6947-10-12 20199680 PMC2845091

[B42] Sheridan SL Golin C Bunton A Lykes JB Schwartz B McCormack L Driscoll D Bangdiwala SI Harris RP Shared decision making for prostate cancer screening: the results of a combined analysis of two practice-based randomized controlled trials BMC Med Inform Decis Mak 2012 12 130 10.1186/1472-6947-12-130 23148458 PMC3582602

[B43] Dear RF Barratt AL Askie LM Butow PN McGeechan K Crossing S Currow DC Tattersall MH Impact of a cancer clinical trials web site on discussions about trial participation: a cluster randomized trial Ann Oncol 2012 23 7 1912 1918 10.1093/annonc/mdr585 22258366

